# One Year Follow-Up of a 4-Year-Old Caucasian Girl Diagnosed with Stage IV Grade C Localized Periodontitis

**DOI:** 10.3390/jcm13164878

**Published:** 2024-08-18

**Authors:** Radu-Andrei Moga, Cristian Doru Olteanu

**Affiliations:** 1 Department of Cariology, Endodontics and Oral Pathology, School of Dental Medicine, University of Medicine and Pharmacy Iuliu Hatieganu, Str. Motilor 33, 400001 Cluj-Napoca, Romania; 2 Department of Orthodontics, School of Dental Medicine, University of Medicine and Pharmacy Iuliu Hatieganu, Str. Avram Iancu 31, 400083 Cluj-Napoca, Romania; olteanu.cristian@umfcluj.ro

**Keywords:** juvenile aggressive periodontitis, periodontal pocket, bone loss, hypophosphatasia, diagnosis, deciduous dentition

## Abstract

Stage IV grade C localized periodontitis (pre-puberal localized aggressive periodontitis/LPP), an extremely rare form of periodontal disease, occurs in otherwise healthy individuals (no signs of dental plaque/calculus) due a hyper-aggressive auto-immune response to high periodontopathic bacteria levels. **Methods**: A 4-year-old Caucasian girl with unusually high mobility of the deciduous lower left canine and localized gingival inflammation was misrecognized by multiple clinicians (initially diagnosed with hypophosphatasia, genetic and metabolic disorders, all turning negative), over a period of 4–6 months, despite initial radiographs showing clear pathognomonic signs. The LPP diagnostic was made by the last clinician, but by then the tooth was lost. Similar inflammation signs appeared around the lower deciduous right canine. X-ray examination showed similar bone and periodontal loss as previously seen, while periodontopathic bacteria tested highly positive. The patient received both mechanical cleaning and ten days of systemic antibiotic treatment (Augmentin and Metronidazole). **Results**: Two months later, inflammation signs disappeared, with periodontal regeneration radiologically present, and only small periodontopathic bacteria precursor concentrations. **Conclusions**: Despite initial periodontal loss, an adequate treatment can keep under control an LPP disease. Moreover, bone and periodontal regeneration appears if periodontopathic bacteria scores are kept lower, showing the importance of fast adequate diagnostic and treatment.

## 1. Introduction

One of the rarest forms of inflammatory periodontal disease is the Stage IV grade C localized periodontitis of small children (i.e., pre-puberal localized aggressive periodontitis-LPP/localized juvenile periodontitis), which usually manifests in clinically healthy individuals [1]. A prevalence of 0.06% for white Europeans is cited [2], but without any reference to the patient’s age (which clinically is of extreme importance since both treatment and communication difficulties are related to the young age). This form of periodontal disease has been reported to affect young individuals (without reporting any age interval), with an extremely rapid progression of bone and periodontal loss (i.e., a “U”-shaped bone loss radiological aspect, which appears in 2–4 months [2,3,4,5,6]) affecting mostly the first molars and incisors [2,3,7,8,9,10,11,12,13].

The onset is insidious in most of the patients, and the younger they are, the lower the chance of it being noticed and therapeutically intercepted [1]. The children complain of some discontinuing minor or mild pain/discomfort lasting a few weeks up to three to four months. After this period, the parents/children, when brushing the teeth, begin to observe a limited mobility of the affected tooth. At close inspection the young patient shows almost no/low oral plaque deposits or inflammation signs (i.e., pathognomonic signs) around or surrounding the affected tooth/teeth [1]. In such situations, the radiological examination is rarely performed; thus, most of the periodontal loss is missed. In a short period of time (usually a few months), the tooth mobility becomes advanced (i.e., tooth is lost), the periodontal destruction is clinically visible, and the window of opportunity for conservative treatment is missed. To the best of our knowledge, there are only a few cases reported in the literature (mostly for adolescents) and none for pre-puberal children [1].

The main problem regarding the treatment of this form of periodontitis is related to the correct diagnostic, since the rarity of the disease, the lack of clinical signs and difficulties in communication with young patients cause most of the clinical practitioners to dismiss the case. That is why most of them pass unnoticed or misrecognized (mistaken for autoimmune, metabolic, endocrine diseases, etc.) [1,7,14]. It has been reported to affect both dentitions (i.e., pre-puberal periodontitis affecting the first primary molars; juvenile periodontitis affecting the first permanent molars and incisors) [2], seeming to confirm the genetic familial aggregation [8]. Nevertheless, other teeth can also be involved (e.g., primary canine) [1].

Periodontitis is a multifactorial disease, with some of the mechanisms being still a debatable subject [1,2,15,16]. Most cases of prepuberal periodontitis have related systemic diseases that produce immunodeficiency with effects over the host response to the microbial plaque [15,16]. There are two types of prepuberal periodontitis, the generalized form due to systemic disease called leukocyte adhesion deficiency and the localized form (LPP) with a still uncertain genetical and multifactorial origin [15,17]. LPP form is also multifactorial, with the main mechanism being an abnormal/hyper-immune response to periodontopathic bacteria and showing familial aggregation [6,7,9,14,18]. Usually there is a symbiotic relationship between the oral biofilm and bacterial community [14]. However, for some reasons (not yet identified, supposedly genetic predisposition and/or environmental factors [8]), an imbalance occurs, leading to an aggressive auto-immune response resulting in bone and periodontal loss due to disruptions of the bone metabolism [1,7,14]. The abnormal/hyperimmune response is triggered by both the plaque biofilm (pathogenic bacteria embedded in extracellular polymeric substance with increased resistance to microbial agents and immune defense) and planktonic counterparts (dispersed pathogenic bacteria with higher virulence) [6,19,20]. Nevertheless, the local oral environment highly influences the bacterial virulence, but the rapid changes in nutrients, oxidative stress and mechanical disruptions of the oral plaque are difficult to study and not yet fully understood [1,6,19,20,21]. Some studies reported *Fusobacterium nucleatum* (less periodontal loss if levels are reduced), *Porphyromonas gingivalis*, *Prevotela intermedia*, *Treponema denticola* and *Tannerella forsythia* as highly periodontopathic bacteria [17,19,21]. Other studies reported also *Actinobacillus actinomycetemcomitans* and *Porphyromonas gingivalis* to trigger the hyper-immune response [8,17,21]. Blood tests also reported higher levels of B lymphocytes in LPP patients [1,7,22]. Thus, the importance of performing both bacterial and blood tests when assessing LPP cases is obvious [1].

In LPP patients due to genetic predisposition [1,17,18], the hyper-immune response is quick, aggressive and disproportionate, with severe localized inflammation and tissular resorption with significant and visible effects in a very short period of time [7,18,22,23]. This excessively aggressive hyperimmune response is triggered not only by periodontopathic bacteria levels but also by currently unknown environmental factors [1,7,14,18,23].

The differential diagnosis for the prepuberal periodontitis is usually made with systemic disease that induces immunodeficiency (e.g., leukocyte disorders, neutropenia, HIV, Chediak-Higashi syndrome, Leukocyte adhesion deficiency syndrome, Papillon-Lefevre syndrome, Down’s syndrome, diabetes mellitus, Hypophosphatasia/Rathbun-syndrome, Histiocytosis syndromes, Ehlers-Danlos syndrome, Juvenile hyaline fibromatosis of gingiva, Virus-associated hemophagocytic syndrome, Malnutrition) [15,16].

Before starting the therapy, the generalized or localized form must be identified. Thus, if no known diseases or immunodeficiency are recorded, common blood count and urine tests are usually performed to see if some disorders are present. Afterwards, if any suspicions are raised, those are closely investigated by using more specific tests. Nevertheless, when clinical examination of the patient is performed, it should be accompanied by X-rays (panoramic and retro-alveolar) and a periodontopathic bacteria test (to identify if they are present, and the bacterial amount). All these previously mentioned data must be placed in the oral context (i.e., localized bone loss and periodontal inflammation) to have a starting point for the diagnosis (i.e., advisable to start from basic to complex in approaching the pathology).

The treatment of LPP patients is challenging, especially due to their young age and difficulties in communicating with them and rapid tissular destruction progression [1]. The aims are to stop the periodontal loss and conserve the tooth/teeth and regain the bone and periodontal ligament loss, to reduce the periodontopathic bacterial levels in order to reduce the hyper-immune response and regain the oral bacterial balance [1,3,8,10,24]. The most important aspect of the treatment is to establish the correct diagnosis based on both clinical and radiological examination and periodontopathic bacterial and blood tests [1,8,10,24,25,26]. The LPP treatment (as for the most of periodontal disease forms) is mostly conservative (scaling, root planing, associated with systemic antibiotics) due to the young age of the patients [1,3,8,10,13,24,26,27]. The most effectively used antibiotic association is metronidazole and amoxicillin combinations, showing better results with systemic than topical administration [1,3,8,10,13,24,27,28]. The “24 hours full-mouth disinfection” concept also improved the clinical prognostic [3,11].

The most common LPP misdiagnoses are made with genetic and metabolic disorders, due to the young age of the patients, the poor objective symptoms (i.e., especially in the early stages of the LPP disease), the rarity of disease and a lack of clinical experience [15,16]. Nevertheless, if properly investigated (radiological examination, periodontopathic bacteria test, and blood count), all the elements necessary for limiting the clinical diagnosis begin to appear [1]. Among bone metabolic diseases, hypophosphatasia is one of the common candidates for the misrecognition of LPP. It has a low prevalence of 1 to 300,000 in Europe being caused by the deficiency in alkaline phosphatase activity and ALPL gene mutation, involving the calcium and phosphate metabolism [15,16,29]. Two main forms are reported to be odonto-hypophosphatasia and systemic hypophosphatasia, both related to defective mineralization of bone due to lack of calcium fixation despite normal absorption [29,30]. The odonto-hypophosphatasia displays dental abnormalities, premature loss of primary teeth, dental caries, reduced dentine thickness and reduced alveolar bone, associated with low levels of parathyroid hormone (due to hypercalcemia and hypercalciuria that would lead to the development of hyperphosphatasemia) [29,30].

Herein, the aim was to report the evolution without and with adequate therapy, of a 4-year-old Caucasian girl’s case, with misrecognized LPP (for half year), localized advanced periodontal loss around the lower deciduous canines, poor objective symptoms and atypical localization.

## 2. Materials and Methods

Herein is an unusual case (given both the young age and the involved tooth) of a 4-year-old healthy Caucasian girl with a known history of familial periodontal problems (manifested especially in the grandparents’ generation) but with no related systemic diseases that induce immunodeficiency. The first subjective and objective symptoms were poor, appearing in the early months of 2023 (February–March), with complaints of small to moderate pains in various places of the oral cavity, lasting a few minutes. After a quick intra-oral clinical examination, the local dentist (i.e., a small suburb of M., Germany) dismissed the case since nothing abnormal was seen. The pain complaints continued, and in June the family observed that the lower left deciduous canine (i.e., 7.3) began to move. The parents returned to the local dentist who, after another quick clinical examination, sent the case to M. University Hospital (Periodontology Department, Germany) [1].

**Figure 1:**
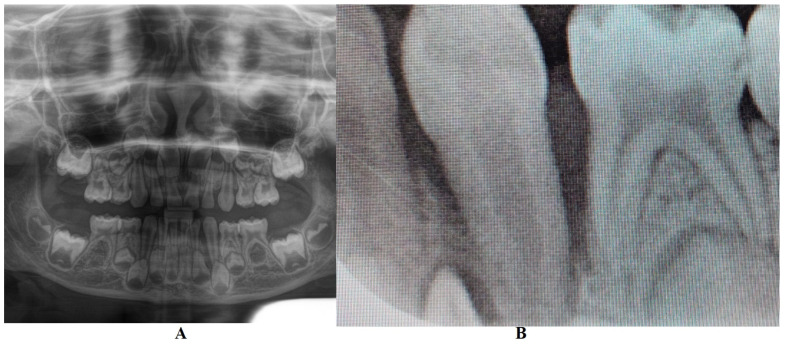
First X-ray radiological investigations (14 June 2023): (**A**) panoramic with advanced periodontal loss around lower left canine, (**B**) retro-alveolar aspect of the 7.3 periodontal loss (München, Germany).

In M. University Hospital (Periodontology Department, Germany, 14 June 2023), a full oral clinical and paraclinical examination was performed including X-ray examinations (retro-alveolar and panoramic) (Figure 1). As a result, the deciduous left canine movement and a localized bone loss around the above-mentioned tooth were confirmed, with suspicion of a metabolic disease (i.e., hypophosphatasia/hyperphosphatasia), while the pain complaints were cataloged as of unknown cause. No other problems were identified (e.g., orthodontic disorders, hyper-eruption, etc.). The case was dismissed with no prescribed therapy, no clinical diagnosis, and only common oral hygiene instructions. The recommendations were to continue the investigations in the Endocrinology Department for blood count and genetic tests [1]. No periodontopathic bacteria test (that would significantly help in setting up the diagnosis) were proposed to the family, despite a clear picture of a localized periodontal disease in a pre-puberal patient (based on clinical examination and patient’s history).

In the Endocrinology Department (M. University Hospital, Germany, 19 June 2023), the blood count test showed (20th of June) only a slight increase in monocytes and lymphocytes and a decrease in neutrophile granulocytes, all other constituents being within age-specific physiological parameters (disconfirming the initial hypophosphatasia suspicion). The urine test was negative (21 June 2023). The genetic tests (taken 17 June 2023, received by parents in late September 2023), were negative, disconfirming the initial suspicion of hypophosphatasia [1].

**Figure 2:**
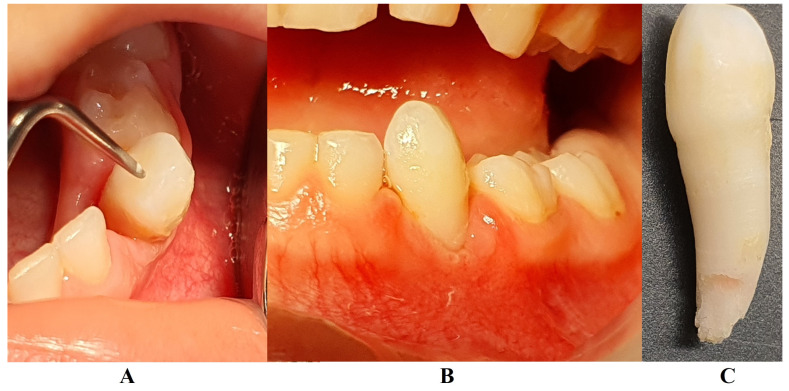
Images of the lower left canine: (**A**) and (**B**)—advanced mobility and hyper-eruption due to advanced periodontal loss and inflammation (2 August 2023), (**C**)—7.3, lost in September 2023.

In August 2023, the family consulted another clinician abroad (2 August 2023, Klausenburg/Cluj-Napoca—Romania) since the parent’s concerns related to the deteriorating local conditions (inflamed periodontium around lower deciduous canine and enhanced movement, Figure 2). The parents presented the entire documentation gathered from previous examinations including paraclinical test results and X-rays [1]. After reviewing the familial history and clinical and paraclinical examinations (3rd of August), the initial hypophosphatasia/hyperphosphatasia suspicions were dismissed (also confirmed by late September 2023 through negative genetic test results), and the Stage IV grade C localized periodontitis/pre-puberal localized aggressive periodontitis-LPP diagnosis was made. No orthodontic problems were detected during the clinical examination (also confirmed by previous clinical exams), and only the hyper-eruption of the deciduous left canine due to advanced periodontal loss and surrounding inflammation (Figure 2) was noted [1]. When setting up the LPP diagnosis, the previous X-rays (Figure 1) were taken into consideration along with the young patient’s other paraclinical results. For laboratory confirmation, a periodontopathic bacteria sample was taken (3rd of August). Due to the advanced localized periodontal loss and tooth movement, only professional hygiene and adjuvant topical applications with amoxicillin and metronidazole association around the inflamed periodontal pocket for a period of 10 days were prescribed. The parents were informed about all these above-mentioned issues, and the most likely loss of the tooth but also the necessity of instituting a general antibiotic treatment and follow-up of the case. The family left Klausenburg (Romania) 5 August 2023. The periodontopathic bacteria test was highly positive on 16 August 2023 with *Fusobacterium nucleatum*/*periodonticum* and Capnocytophaga. The tooth was finally lost in early September (Figure 2) [1].

In late October 2023 (i.e., 23 October), the deciduous lower right canine (i.e., 8.3) showed a localized inflammation of the surrounding periodontium and mobility. They consulted one more time the local dentist (i.e., a small suburb of München, Germany), who confirmed the localized periodontal inflammation around 8.3 and its mobility, but also mobility signs of 5.3, deciduous upper right incisors (i.e., 5.2 and 5.1), and deciduous lower incisors (i.e., 8.1, 8.2, 71, and 7.2) but no inflamed periodontium. The recommendation was antibiotic treatment with Augmentin of 200 mg (600 mg/day, one spoon three times/day) for 10 days, and one week after the treatment ceased, a periodontopathic bacterial test was taken, that came with negative results.

**Figure 3:**
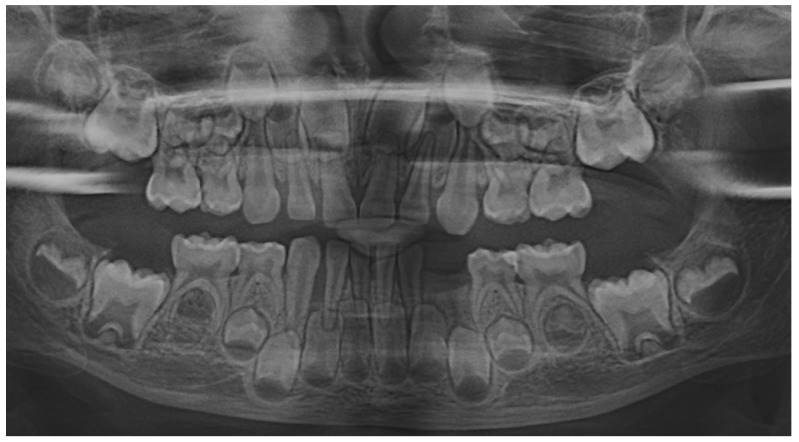
Panoramic X-ray radiological investigations with advanced periodontal loss around the deciduous lower right canine, and periodontal gain around deciduous lower left canine site (late December 2023) (Klausenburg, Romania).

**Figure 4:**
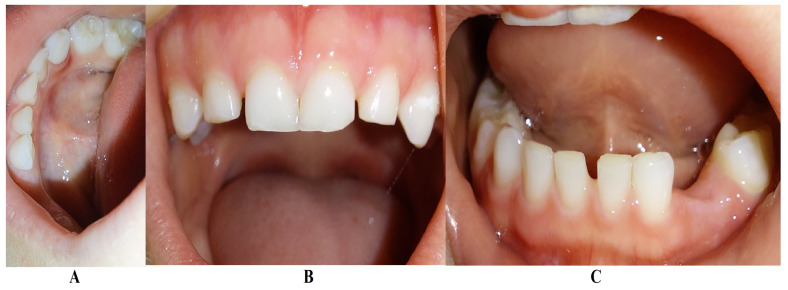
Images of the oral status in late December 2023: (**A**) 8.3. localized lingual gingival inflammation, (**B**) maxillary teeth, (**C**) lower left canine site with signs of periodontal gain.

The family kept in touch with the clinician who diagnosed the LPP disorder informing them about the case evolution, and in late December 2023, the family returned to Klausenburg (Cluj-Napoca—Romania). The panoramic X-ray (Figure 3) confirmed not only the presumed advanced periodontal loss around 8.3 but also bone loss around the upper deciduous canine and incisors (i.e., 5.3, 5.2 and 5.1), when compared with the previous panoramic from June 2023 (Figure 1). Another investigated aspect was the lower left region of previous troubles, with no radiological signs of definitive canine sufferance (i.e., 3.3) and visible signs of bone regeneration. The oral clinical examination confirmed the lack of dental plaque and calculus, the advanced mobility of the deciduous lower canine, a light mobility of the other above-mentioned teeth and healing of the lost deciduous lower left canine area (Figure 4). Another periodontopathic bacterial test was taken to reflect the latest bacterial types. Nevertheless, an oral professional cleaning was performed and based on previous LPP positive diagnosis, positive periodontopathic bacterial test and clinical evolution, a systemic antibiotic treatment was prescribed. The prescribed antibiotic (girl’s weight was about 15 kg) association was of Augmentin of 500 mg (1 g/day, 1 table twice a day) and Metronidazole of 250 mg (500 mg/day, 1 tablet twice a day, but with the need to adjust the dosage if allergy/overdose signs were to be displayed) for a period of 10 days and a periodontopathic bacterial test 2–3 months after the treatment ceased.

The family returned home and the young patient (who had turned 5 years old by now) started the antibiotic treatment immediately in early January (i.e., 8 January) 2024, remaining in contact with the Klausenburg clinician. Metronidazole daily dosage (despite being at the upper limit) induced vomiting after the second day (i.e., 10 January), and it was decided to reduce by half the daily dosage to 250 mg/day (from initial 500 mg/day), fragmented in two (125 mg twice a day, taken together with the Augmentin dose). After this dosage adjustment no other incident appeared, finishing the treatment on the 18 January.

The periodontopathic bacterial test taken before the antibiotic prescription (i.e., late December 2023) was highly positive with five types of bacteria (when compared with only two types in the previous test received 16 August 2023). Thus, besides the two types present in first test (i.e., 16 August 2023), *Fusobacterium nucleatum*/*periodonticum* and Capnocytophaga already present in the oral cavity (and responsible for the loss of 7.3 and localized periodontal destruction), remaining highly positive, another three types showed up mildly positive: *Treponema denticola*, *Eubacterium nodatum* and *Eikenella corrodens*, confirming not only the clinical worsening of the oral condition (Figure 3 and Figure 4) seen during oral examination (late December 2023) but also the chosen antibiotic association scheme.

Meanwhile (18 January) the immunology test was (taken 23 November 2023) positive with high levels of IgE (i.e., 828 U/mL when physiological normal is below 60 U/mL) and a light increase for CRP/C-Reactive Protein (i.e., 1.2 mg/dl instead of 1 mg/dl), reticulocytes (i.e., 17 Promille instead of 15 Promille), MPV (11.5 ft instead of 11 ft), eosinophile granulocytes (4% instead of 3%) and monocytes (0.9 G/l instead of 0.77 G/l), confirming once more the initial diagnosis of LPP (localized pre-puberal periodontitis).

## 3. Results

**Figure 5:**
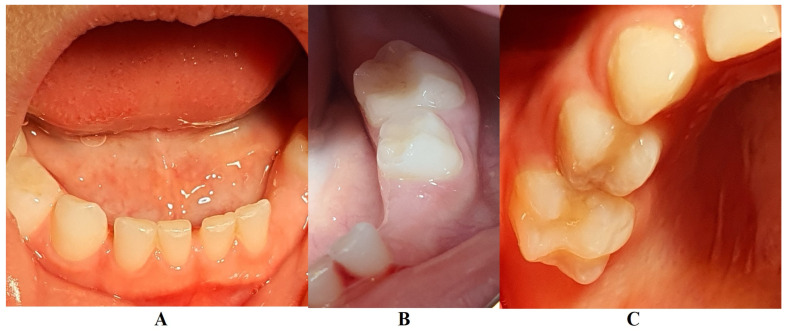
Images of the oral status in May 2024: (**A**) lower right canine site, (**B**) lower left canine site, (**C**) upper right canine site.

**Figure 6:**
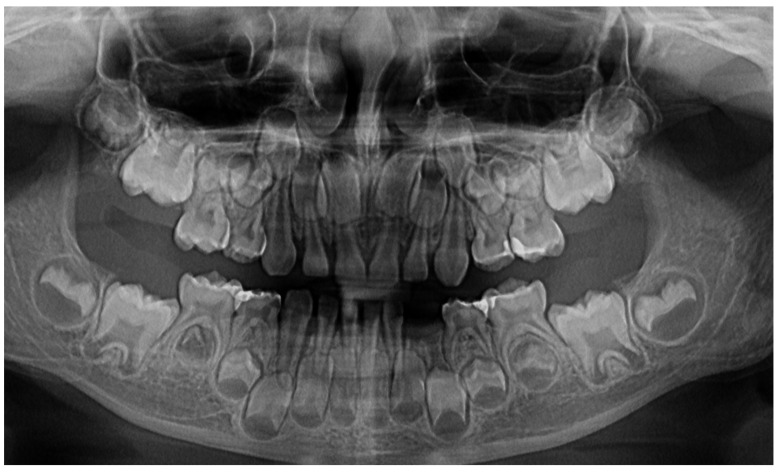
Panoramic X-ray radiological investigations with visible signs periodontal gain around deciduous lower right canine (May 2024) (Klausenburg, Romania).

In early May 2024 (i.e., 15 May 2024) the family returned to Klausenburg (Cluj-Napoca—Romania), for a three-month control and evaluation. The clinical examination showed physiological mobility of all previously involved teeth, with no signs of periodontal inflammation and no plaque/calculus (Figure 5). No reports of pain complaints were reported (The last one being reported in October–November 2023). The panoramic radiological examination (Figure 6) showed a spectacular and visible periodontal regeneration and gain around 8.3 (that previously, in late December 2023, showed high periodontal loss), with visible periodontal gain around the upper and lower incisors. The definitive teeth showed physiological normality (especially the 3.3, closer to the periodontal loss site). A periodontopathic bacteria test was also taken (17 May 2024). The results (31 May 2024) revealed the presence of only “precursor germs of highly pathogenic ones” but with “no detectable highly pathogenic germs”. Nevertheless, the revealed precursor germs were those of *Fusobacterium nucleatum*/*periodonticum*, *Campylobacter rectus*, *Eubacterium nodatum*, *Eikenella corrodens* and *Capnocytophaga* spp. The results at three months were considered to be favorable, meanwhile a 6 months’ control was scheduled for the autumn of 2024. However, up to early August 2024 (when the report herein was written) no LPP reactivation signs showed up and no more pain complaints were reported.

## 4. Discussion

The report herein describes a case of Stage IV grade C localized periodontitis (pre-puberal localized aggressive periodontitis/LPP) in a 4-year-old Caucasian girl, as well as its evolution over a period of one and half years (i.e., early January 2023 up to early August 2024). No similar case was found in the current research flow [1].

The unusualness of this case is due to several aspects. The first is related to the extremely young age of the patient (i.e., 4 years old in February 2023, deciduous dentition), with an insidious onset, and apparently no familial aggregation or history. The second aspect is related to the misdiagnosis of metabolic diseases (i.e., hypophosphatasia/hyperphosphatasia as written) with oral manifestations (since no therapeutic measures were taken) despite the initial radiographical (i.e., Figure 1) and clinical examination that suggested a clear picture of an unusual LPP (e.g., the lower left canine involvement). The third aspect is related to the lack of therapeutic measures that rapidly set the course for the periodontal and 7.3 loss, in an interval of around 7 months (Figure 2) and the further progression involving other oral sites (Figure 3 and Figure 4). The fourth aspect is related to the evolution under adequate treatment (despite no written report to guide it) and the periodontal gain over a period of around 8 months following the antibiotic therapy (Figure 5 and Figure 6).

The young age of the patient (4 years old) led to a superficial consideration of oral pain reports (for a period of 5 months, February–June), backed up by no clinical elements of concern found by the first dentist who approached the case. However, no mobility tests reported for the deciduous teeth and especially no radiographical examination were taken (even for preventive dentistry reasons) despite the repeated reported pain complaints.

In order to be able to correctly diagnose the case herein, it must be emphasized that LPP is a very rare form of periodontal disease usually with familial aggregation (despite here, where this aspect was negative) [1,2,3,7,8,9,10,11,12,13,24,25,26,27,28]. The pathogenic mechanism relies on a hyper-immune disproportionately aggressive response to periodontopathic bacteria [6,19,20,21] found in the oral cavity that led to rapid bone metabolism disruptions, soon followed by periodontal and tooth loss [1,2,6,7,8,9,10,14,18,22,23]. There is no available treatment of the condition [1]. Moreover, it is not fully understood and recognized (since almost no scientific data are available), and from this point of view the report herein brings new data to clinical treatment field [1]. Since no changes in how the hyper-immune mechanism can be performed (especially in a 4-year-old), the only possibility resides in managing the trigger factor (i.e., the amount of periodontopathic bacteria found in the oral cavity) [1]. To achieve this, the bacterial types and their amount must be investigated.

The clinical examination as well as the complementary radiological one could point out the LPP (localized pre-puberal periodontitis) diagnosis, despite the unusual involvement and limitation to deciduous canines (the literature reports the involvement of molars and incisors) [2,3,7,8,9,10,11,12,13]. The initial “U”-shaped bone loss [2,3,4,5,6] and limitation to the 7.3 site can easily exclude a general metabolic disease with oral manifestations (where usually there is no such limitation). The hypophosphatasia/hyperphosphatasia misdiagnosis (i.e., June 2023) can be easily rejected since the blood count and urine test displayed no such signs (i.e., high phosphate serum levels, low levels of parathyroid hormone due to hypercalcemia and hypercalciuria) [15,16,17,29,30]. The initial blood test (20 June 2023) revealed a slight increase in monocytes and lymphocytes, and a small decrease in neutrophile granulocytes, which are perfectly normal in a case of localized inflammation due to bacterial infection. The general form of prepuberal periodontitis related to systemic diseases (i.e., induce immunodeficiency affecting the response to the microbial plaque [15,16]) seemed not to be considered when the first clinical examination was performed (June 2023) even though hypophosphatasia is among the conditions with which the differential diagnosis can be made of.

The rapid periodontal loss which characterizes the LPP means that the treatment window of opportunity is very tight. In this case a period of 5 months was lost, with no diagnosis and no treatment, which lead to rapid periodontal loss and deciduous left canine loss.

The only practical approach in such cases is to address the triggering factor, i.e., the large number of bacteria that trigger the aggressive hyper-immune response [6,19,20,21]. Thus, a simple periodontopathic bacteria test can easily confirm the LPP diagnosis (as it did here, in August 2023). Based on the bacterial type, a proper antibiotic association can be established (usually amoxicillin with metronidazole) [1,3,8,10,13,26,28]. Nevertheless, due to the young age of the patient, the dosage must be approached with care. Another aspect that needs to be addressed with elegance and tact is communication with the parents, because of the previous negative experiences in dealing with the case (i.e., varying degrees of mistrust). A rational scientific evidence-based approach might help.

The anticipated progression of LPP under the lack of treatment and its consequences must be communicated to the family (as here, in August 2023, and unfortunately correctly anticipated) since the legal consequences could be significant. Nevertheless, the treatment must also be based on scientific data and with complete understanding of the pathogenic mechanisms (i.e., previous incorrect prescription both in terms of antibiotic type and dosage, October 2023, Augmentin 600 mg/day). The periodontopathic bacterial test recommendation (to be repeated after a period of two–three months after the antibiotic ceases) has a strong scientific point and must be followed. If repeating the test a week after the antibiotic is stopped (as incorrectly was performed), the test will only show a biased result (i.e., as it did herein for the October 2023 test).

The natural periodontal gain observed in this case is important from the prognosis point of view. It was observed that the site of the lost 7.3 showed periodontal gain (i.e., Figure 3, the panoramic form early January 2024), in the absence of the correct antibiotic association (since solely Augmentin did not cover all pathological bacterial types). However, it must be emphasized that one cannot predict the impact on this matter of the above-mentioned incorrect antibiotic treatment performed in October 2023 (i.e., no signs of other periodontal gain on the upper and lower incisors and only progression of the periodontal loss around the 8.3). Nevertheless, significant periodontal gain was observed on the last panoramic (Figure 6, May 2024) after the correct antibiotic association.

The aim of the treatment was only to minimize the triggering factor (the bacterial amount) of the aggressive hyper-immune response in order to minimize the periodontal loss of LPP (which was achieved) and to maintain it for a variable period of time (in order to buy time). To completely remove the periodontopathic bacteria in this case seems to be not possible (as the evolution showed) both due to the unknown nature of the LPP disease, mechanism, bacterial origin and proliferation and not last the lack of knowledge related to this subject.

## 5. Conclusions

1. The rapid diagnosis of LPP is essential in order not to miss the treatment window of opportunity to preserve/minimize the periodontium/periodontal loss.
2. The diagnosis should be essentially based on the clinical and radiological examination and supported by paraclinical testing. Moreover, repeated pain complaints (even from a 4-year-old) should be taken seriously and not easily dismissed.
3. The periodontopathic bacterial test is essential to have a workable antibiotic association protocol, as well as assessing the LPP progression.
4. The periodontal gain herein proves that even at a young age under adequate treatment, LPP can be kept under control, providing correct and physiological development from both orthodontic and periodontic points of view.

## 6. Clinical and Scientifical Significance

Due to the rarity of the Stage IV grade C localized periodontitis/pre-puberal localized aggressive periodontitis-LPP (0.06%) and the difficulty related to following the clinical case, there are no available data in the current research flow about pre-puberal cases. Moreover, little is known regarding the adequate workable treatment (for pre-puberal patients) as well as the evolution with and without treatment. Another aspect is related to the knowledge needed to establish the correct clinical diagnosis. Herein is the first report to addresses the above-mentioned issues, assessing the evolution of a 4-year-old Caucasian girl with LPP (misdiagnosed with metabolic disease for almost half a year), with periodontal loss due to lack of proper treatment for about a year, and then the favorable evolution with correct treatment for more than 8 moths.

## Data Availability

All needed data are displayed herein.
